# Mental Suggestion as an Aid in the Treatment of Morphinomania

**Published:** 1895-05

**Authors:** Sam’l H. Green

**Affiliations:** Oakdale, Ga.


### MENTAL SUGGESTION AS AN AID IN THE TREATMENT OF MORPHINOMANIA.*

BY SAM'L H. GREEN, M.D.,
OAKDALE, GA.

I shall not attempt to elucidate the mysteries of psychic phenomena, nor will I venture upon a discussion of suggestion which has been called the all-powerful factor in hypnotism, but rather beg leave to offer for your consideration a résumé of two cases of morphinomania which I successfully treated and in which I was greatly aided by suggestion.

On September 10, 1893, I received into the hospital of the Chattahoochee Brick Company's branch of the Georgia penitentiary Robert D., white, age thirty-five, who had been, according to his own statement, addicted to the morphine habit for over five years. The prison guard who brought him from the jail, not being instructed by the county physician, refused to let him have the drug during the trip, and, as a consequence, when he reached the hospital he was almost immediately seized with a violent convulsion. A letter from a reputable physician, who had attended him professionally during his incarceration in jail, stated that he was in the habit of using four hundred grains of morphine a day. I restored patient by hypodermatic injections, and ascertained that his stomach frequently failed to take up the morphine, and then he was in the habit of resorting to the needle. Hundreds of red blotches, which literally covered his arms and legs, bore out this statement. The patient said that he had been told that when he arrived at the penitentiary, the morphine would be cut off, and begged piteously that it be not done. After reflection I decided upon a new line of treatment in this case, and in order to secure the patient's confidence, I resorted to diplomacy. I told him that he had been misinformed, that his supply would not be cut off, but that he had been addicted to the habit so long that he could not do without it, and that I would furnish him the drug as long as he remained in prison. As an earnest of good faith on my part I gave him a little over three ounces of morphine in a bottle, in-

*Read at late meeting of the Medical Association, Savannah.

structing him to take it when he thought he needed it, and to come to me for another supply when he had used that. Promptly on the afternoon of the fourth day he asked for another supply. In the interim I had kept patient under surveillance, and in every way possible had fortified my already strong hold upon his confidence. I told him that the morphine I had had been on hand for several months, and when kept so long lost a percentage of its potency, and to counteract this loss of strength it would be necessary for me to make a solution and add another drug to the mixture. I took a quart bottle, filled it with water, and put three hundred and sixty grains of morphine in it; of this mixture I gave him a tablespoonful three times a day, and placed him on a light, nutritious diet. On third day patient showed rapid, nervous heart. I told him it would be necessary to increase strength of mixture, and that I would give him a drug which, when taken into the stomach an hour after the morphine solution, would make the latter more effective. In the meantime I had secured a quart bottle exactly like the one containing the morphine mixture, and after filling it with water, had placed it in my medicine cabinet. When I noticed that he observed my actions, I took the bottle of water out, and putting about half an ounce of sulphate of quinine in it, set it away in the cabinet again. The patient smiled confidently when he saw me do this. I then gave him his regular dose of the morphine solution, telling him that the recent addition had made it extraordinarily strong. Patient concurred with me in this, saying that it tasted "awful strong." Patient complained that his legs and arms pained him like they did when he had been deprived of morphine on his journey from the jail to the penitentiary. I told him that it was caused by rheumatism, and that I would give him something that would cure that. I took a two-ounce bottle, put four fluid drachms each of tincture of iron, chloride, and digitalis in it, filled it with water, and commenced giving him ten drops three times a day. I had kept patient on morphine mixture two weeks, when I commenced a further reduction by putting in a tablespoonful of water every time I gave patient a dose of the mixture. I kept him on this treatment, also the digitalis and iron, for four weeks, when I commenced to give him five grains of quinine in solution four times a day. Patient began to improve greatly, circulation became better, and his appetite increased wonderfully. At the expiration of seventy-two days I gave patient last dose of morphine mixture. Under the reduction of one thirty-second of the solution, each dose was virtually water, and had been so several days. Patient continued taking quinine solution, believing he was still on morphine mixture. I kept up digitalis and iron, and in exactly four months from date of entrance to hospital he was doing light work in prison blacksmith shops. Patient weighed one hundred and twenty-eight pounds when he arrived at prison, and six months from date of reception weighed one hundred and ninety-five. When I informed him that it had been some weeks since he had taken any morphine, he was greatly astonished, but after awhile seemed much pleased, saying that he would never use morphine again as long as he lived. Prisoner was pardoned by the governor shortly afterwards, and went back to his home where he now pursues his avocation of blacksmith. I have received several letters from him, in which he says he thanks God that he is free from the habit forever.

Edwin H., white, aged twenty-seven, was admitted to the prison hospital on the 16th of June, 1894. Patient had been an opium smoker for two years and had then shifted to morphine, and had been addicted to the morphine habit over three years. When I first saw him patient was using about three hundred and eighty grains of morphine daily; patient had been an inveterate cigarette smoker for over twelve years, and so excessive had been his use of them that on the first day of his admission to the hospital he had two hemorrhages from his lungs, losing nearly half a pint of blood. I stopped hemorrhages by giving elixir of vitriol, one-half ounce, added to four ounces of water in half teaspoonful doses in wineglass of sweetened water every hour. Patient realized that excessive cigarette smoking had caused the hemorrhages and never cared for them again. Patient was a man of intelligence, and I commenced to secure his confidence by telling him that I sympathized with him greatly in his trouble and that I would be pleased to do anything I could for him. Patient said that he had been informed that in penal institutions it was customary for the physician to cut down the supply to morphine habitués in the shortest possible time; said he had been using opium and morphine so long that if he were denied his usual quota his shattered nervous system would not be able to stand the shock, and that he would surely succumb. I concurred with patient and told him that, as he only had to stay in prison twelve months, I would supply him with as much of the drug as he desired. In the interim to counteract the effect of the two hemorrhages I gave him subcutaneous injections of five drops digitalis and half a drachm of whisky. I let him have his usual quota of morphine for four days, after which I made a solution of three hundred and sixty grains of morphine in quart of water as in case number one. I told patient that, on account of the hemorrhages, it would be necessary for me to make a solution of the morphine so that I could incorporate another drug to increase its power. In order to strengthen him, I put him on four drachms of the quinine solution three times a day. I also gave him ten drops of the digitalis and tincture of iron chloride three times a day. On same day reduction of morphine was made patient complained of pains in legs and arms. I gave him hypodermic injections of half a drachm of whisky at intervals of six hours, keeping up the injections for three days, at the expiration of which time I ceased giving the whisky and commenced giving him five grains of quinine sulphate four times a day. In the meantime had placed him on a light, nutritious diet, occasionally between meals giving him a cup of malted milk. Patient inquired if I had not reduced morphine some. I told him that the medicine I had given him for the hemorrhage had blunted his sense of taste, but that if he did not think it strong enough I would add more morphine. I then went through the operation of putting quinine in a quart bottle of water where he could observe the action. Patient was immediately reassured and commenced to mend rapidly. When he had been on morphine mixture two weeks, I commenced reduction by placing in bottle a tablespoonful of water for every one of the mixture taken out. Reduction was gradual, but patient improved, and at the end of sixty-two days I told him he was taking water made bitter by a little quinine. Patient was surprised, but said he was glad that I had adopted that method of reduction. Patient weighed one hundred and twenty-six pounds when admitted to hospital. He at this writing weighs one hundred and eighty-five pounds. He is now, April 1, 1895, hospital steward at the prison hospital, handles morphine almost every day, and is thoroughly cured of the habit. To use his own language, he "becomes thoroughly nauseated when he thinks that he was ever addicted to the habit of using the vile stuff."

The remarkable feature in these cases was the enormous reduction from four hundred grains in case number one and three hundred and eighty grains in case number two in twenty-four hours, to fifteen grains each in both cases in the same length of time without any bad effect except the rapid, nervous heart and pains in the limbs, which disappeared like magic under the combination of digitalis and iron, quinine and suggestion.

Will you not concur with me, gentlemen, when I say that suggestion is a great aid, if nothing more, in the treatment of morphinomania?

I beg leave to say that I am much indebted to Dr. Wm. O'Daniel, Ex-State Physician, for valuable advice in connection with the above cases.

